# The Influence of Interdental Brushes and Toothpaste On Approximal Enamel and Dentine Abrasion – A Laboratory Study

**DOI:** 10.3290/j.ohpd.c_1955

**Published:** 2025-04-15

**Authors:** Chiara Marconi, Andrea Gubler, Florian J. Wegehaupt, Patrick R. Schmidlin

**Affiliations:** a Chiara Marconi Doctoral student, Clinic of Preventive Dentistry, Periodontology, and Cariology, Center of Dental Medicine, University of Zurich, Zurich, Switzerland. Performed the experiments in partial fulfilment of the requirements for a doctoral thesis degree and wrote the manuscript.; b Andrea Gubler Lab manager, Clinic of Preventive Dentistry, Periodontology, and Cariology, Center of Dental Medicine, University of Zurich, Zurich, Switzerland. Developed the experimental design, contributed substantially to the discussion and writing of the manuscript, and proofread the manuscript.; c Florian J. Wegehaupt Prof Dr Med Dent, Head of Division of Preventive Dentistry and Oral Epidemiology, Clinic of Conservative and Preventive Dentistry, Center for Dental Medicine, University of Zurich, Zurich, Switzerland. Developed the experimental design, contributed substantially to the discussion and writing of the manuscript, and proofread the manuscript.; d Patrick R. Schmidlin Prof Dr Med Dent, Head Division of Periodontology and Peri-Implant Diseases, Clinic of Conservative and Preventive Dentistry, Center for Dental Medicine, University of Zurich, Zurich, Switzerland. Conceptualised the idea for the study, developed the experimental design, contributed substantially to the discussion and writing of the paper, and proofread the manuscript.

**Keywords:** abrasion, enamel, dentine, interdental brush substance loss

## Abstract

**Purpose:**

To investigate the approximal abrasive enamel and dentine wear using interdental brushes (IDBs) with and without toothpaste in a novel standardised *in-vitro* set-up.

**Materials and Methods:**

Seventy-two bovine enamel and 72 dentine specimens were prepared and randomly allocated into 12 groups (odd group: dentine; even group: enamel). The specimens were brushed with three IDB types of ISO 2 (Curaprox (CPS09, groups 1–4), Elmex (size 2, groups 5–8) and Circum (Circum 2, groups 9–12)) with artificial saliva or toothpaste slurry (Colgate Total Original). A custom-made brushing device simulated interdental brushing for 1 h on dentine (7,200 strokes) and 6 h on enamel (43,200 strokes). Wear was assessed using a contact profilometer, and electron microscopy images were taken. Kruskal–Wallis and Mann–Whitney tests were used for statistical analysis.

**Results:**

The combination of IDBs with artificial saliva resulted in enamel and dentine wear below the detection limit, similar to the enamel wear when toothpaste was used. Dentine specimens showed significant abrasive wear, which was influenced by the IDBs’ design as follows: Curaprox (median ± interquartile range (IQR): 8.6 ± 1.0 µm), Circum (9.7 ± 2.9 µm), and Elmex (18.8 ± 9.1 µm). The difference in wear between Curaprox and Circum was not statistically significant (P = 1). However, the increase in the wear of Elmex compared with that of the other IDBs was significant (P < 0 0.001).

**Conclusion:**

The use of IDBs with toothpaste may cause statistically significant dentine wear and should not be recommended in combination. Appropriate instructions are essential.

There has been a 90–95% reduction in the prevalence of caries over the past 50 years due to various preventive and prophylactic measures.^23^ Consequently, the clinical focus has shifted toward non-carious hard tissue lesions, particularly in an ageing patient population with retained dentition. Non-carious dental hard tissue loss can occur through mechanisms such as abrasion, erosion, and attrition. Abrasion, caused by mechanical wear from substances other than dental hard tissue, commonly manifests on all dental surfaces, with a higher prevalence at buccal sites.^2,16^ However, atypical lesions in the proximal and lingual-cervical region, resulting from the misuse of oral hygiene products, are increasingly being observed.^10 ^ With the more frequent use of interdental brushes (IDBs) as additional cleaning aids, this issue has gained prominence.

The aetiology of abrasion is multifactorial, involving the quality and abrasiveness of oral hygiene products.^13^ Recent research has highlighted that toothpaste significantly impacts the abrasion of hard tissue, while the toothbrush itself plays a comparatively minor role.^1^ The abrasiveness of toothpaste is measured by the relative enamel abrasion (REA) or relative dentine abrasion (RDA) value, with the latter being the most commonly employed metric.^9^ The American Dental Association (ADA) recommends that toothpaste should not exceed an RDA value of 250 in everyday use.^19^

Despite considerable research on toothbrush abrasion, a notable gap persists in the existing literature regarding the impact of IDBs on dental hard tissue wear. This study aimed to address this gap by investigating the abrasiveness of three different IDBs, with and without the application of an abrasive toothpaste. The null hypothesis was that the use of toothpaste would not lead to significantly higher dental hard tissue loss and that different IDBs of the same ISO size would not be influential in this regard.

## MATERIALS AND METHODS

### Specimen Preparation

Seventy-two enamel and 72 dentine specimens were obtained from the bovine incisors of 40 animals aged approximately 1.5 to 2 years. Six to eight incisors were collected from each jaw and placed in a box from which they were randomly selected in no systemic order. Six samples were obtained from each tooth.

Cylindrical specimens were obtained from the roots and crowns of the bovine teeth using a cylindrical diamond-coated trephine mill with a 4-mm-internal-diameter, with constant water cooling maintained during the procedure. All specimens were embedded in acrylic resin (Paladur, Heraeus Kulzer, Hanau, Germany) in a 6-mm-internal-diameter silicone template. The acrylic resin was polymerised at a temperature of 55°C and a pressure of 2 bar for a period of 10 min in a laboratory polymerisation unit (Palamat Elite, Heraeus Kulzer). To obtain standardised surfaces of the specimens and remove the outer layer of the cementum, the specimens were polished in an automatic grinding machine using 2,000- and 4,000-grit carborundum paper (Tegramin-30, Struers; Copenhagen, Denmark) at a pressure of 1 N for 30 s under continuous water cooling. Two parallel grooves were carved in acrylic resin on each side of the specimen to create reference points for subsequent profilometric measurements.

It is important to note that the animals in this study were reared and slaughtered for commercial food production in accordance with Swiss animal welfare guidelines. The study design did not affect the animals’ lives or the slaughter process. Consequently, this was not considered an animal study, and the protocol was not opposed by the institutional ethics committee.

### Brushing Procedure

A comprehensive overview of the experimental design and allocation is presented in Table 1. Six specimens obtained from each tooth were allocated across six dentine or enamel groups, with each group consisting of 12 specimens (odd groups consisting of dentine and even groups of enamel specimens).

**Table 1:** Study design

72 enamel and 72 dentine specimens from bovine roots (4 mm)
Specimen preparation and standard surface treatment
Recording of baseline profiles (profilometer)
IDB 1: Curaprox	IDB 2: Elmex	IDB 3: Circum
Group 1 Dentin n = 12	Group 2 Enamel n = 12	Group 3 Dentin n = 12	Group 4 Enamel n = 12	Group 5 Dentin n = 12	Group 6 Enamel n = 12	Group 7 Dentin n = 12	Group 8 Enamel n = 12	Group 9 Dentin n = 12	Group 10 Enamel n = 12	Group 11 Dentin n = 12	Group 12 Enamel n = 12
Artificial saliva	Colgate Total Original	Artificial saliva	Colgate Total Original	Artificial saliva	Colgate Total Original
Brushing sequence (enamel 6 h, dentine 1 h, 120 strokes/min)
Recording of final profiles (profilometer) and scanning electron microscopy


Three ISO 2 normed IDBs with different designs were selected for this study: Curaprox (CPS 09 (Curaden, Kriens, Schweiz); groups 1–4), Elmex (size 2 (GABA Schweiz, Therwil, Switzerland); groups 5–8), and Circum (Circum 2 (Top Caredent, Zurich, Switzerland); groups 9–12). While the Curaprox and Elmex IDBs had cylindrical shapes, Circum was waist-shaped. Mechanical treatments were performed on the enamel and dentine using either artificial saliva (groups 1, 2, 5, 6, 9, and 10) or toothpaste slurry (groups 3, 4, 7, 8, 11, and 12). The toothpaste slurry was composed of Colgate Total Original (Colgate-Palmolive, Therwil, Switzerland) and modified artificial saliva, based on the original formulation used by Klimek et al, at a ratio of 2:1.^15^ The composition and constituents of the artificial saliva mixture are outlined in Table 2.

**Table 2:** Constituents of artificial saliva mixture

Position	Chemical	Formula	MG	mmol/l	Liter	1
1	Ascorbic acid	C_6_H_8_O_6_	176.13	0.0114	g	0.002
2	D+glucose	C_6_H_12_O6	180.16	0.167	g	0.03
3	Sodium chloride	NaCl	58.44	9.92	g	0.58
4	Calcium chloride dihydrate	CaCl_2_ ● 2H_2_O	147.02	1.530	g	0.225
5	Ammonium chloride	NH_4_Cl	53.49	2.99	g	0.16
6	Potassium chloride	KCl	74.55	17.0	g	1.27
7	Sodium thiocyanate	NaSCN	81.07	1.97	g	0.16
8	Potassium dihydrogen phosphate	KH_2_PO_4_	136.09	2.42	g	0.33
9	Urea	CO(NH_2_)_2_	60.06	3.33	g	0.2
10	Di-sodium hydrogen phosphate	Na_2_HPO_4_	141.96	2.40	g	0.34


Brushing was performed using a novel custom-made brushing device, as previously described.^21^

The enamel specimens were brushed with 120 vertical brushstrokes per min for 6 h, resulting in a total of 43,200 strokes. The dentine specimens were brushed at the same speed for 1 h, resulting in 7,200 strokes.

### Wear Measurement

A contact profilometer (Perthometer S2, Mahr; Göttingen, Germany) was employed to ascertain the surface profiles of the specimens and calculate the respective abrasive wear of the enamel and dentine.

Five parallel surface profiles, each 4.8 mm in length, were recorded 250 µm apart. A prefabricated template was used for accurate repositioning of the specimen in the profilometer. For each specimen, baseline surface profiles were obtained before treatment, followed by corresponding post-treatment measurements. Tooth substance loss was calculated by superimposition using custom 4D software (4D Deutschland, Eching, Germany). The detection limit and the potential measurement error of the method were found to be 0.1 µm. To avoid bias, the investigator who performed the wear assessment was blinded.

Additionally, electron microscopy images of the specimen surfaces were obtained after brushing. Reference specimens, one enamel and one dentine, were prepared to distinguish notches that could potentially be caused by polishing. The specimens were polished but not brushed. To prepare the electron microscopy images, the specimens were dehydrated using an ethanol series.

One specimen was randomly chosen from each group and prepared for the scanning electron microscope (SEM). The selected specimens were affixed with a carbon pad to an SEM carrier (coloured at the edge with Leit C) and spattered with 10 nm gold. Images were taken at 10 kV and 200 pA using a Zeiss Gemini SEM 450 microscope (Jena, Germany).

The data set is available on request from the authors.

### Statistical Analysis

The data were coded and documented using Excel (version 16.70, Microsoft, Redmond, Washington, USA) and statistically analysed using DATAtab Team (2022) (DATAtab: Online Statistics Calculator. DATAtab, e.U. Graz, Austria, URL https://datatab.net). Descriptive statistics were used to describe means, medians, standard deviations, and interquartile ranges (IQR), descriptive statistics were employed. Non-parametric Kruskal–Wallis and Mann–Whitney tests were used to assess significant differences between groups. A significance level of P < 00.05 was defined for all statistical tests. To ensure the objectivity of the results, the individual responsible for the statistical analysis was blinded.

## RESULTS

### Abrasive Enamel and Dentine Wear

The profilometric results are summarised in Table 3.

**Table 3:** Results of the profilometric measurements of substance loss in μm (n = 12 per group)

	Median value	Interquartile range
Enamel	Curaprox Saliva	0	0
Elmex Saliva	0	0
Circum Saliva	0	0
Curaprox Colgate	0	0
Elmex Colgate	0	0
Circum Colgate	0	0
Dentin	Curaprox Saliva	0	0
Elmex Saliva	0	0
Circum Saliva	0	0
Curaprox Colgate	8.6	1.0
Elmex Colgate	18.8	9.1
Circum Colgate	9.7	2.9


**Fig 1:**
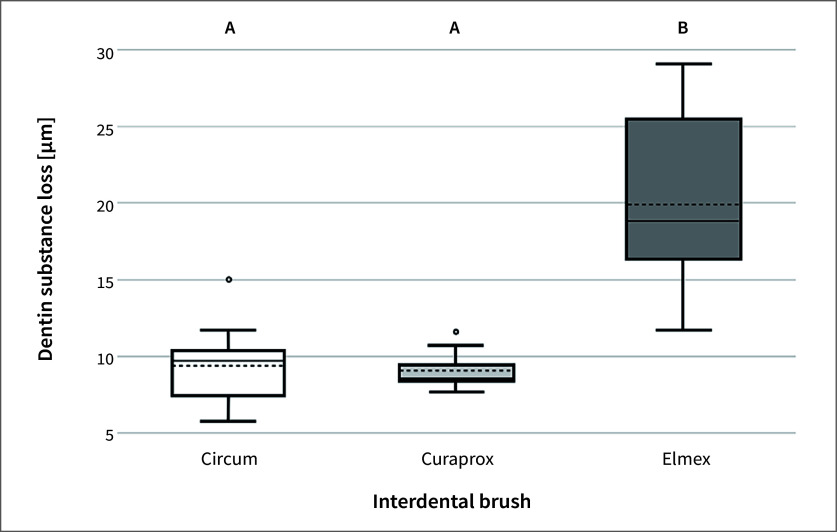
Boxplot illustration depicting the dentine abrasion in µm of the different IDBs with the application of toothpaste after 1 h of brushing (equivalent to 7,200 strokes). Different capital letters indicate statistically significant differences (assessed by Kruskal–Wallis and Mann–Whitney tests; P < 0.05). In the boxplot, the dotted lines mark the mean values, and the continuous lines indicate the median values.

The abrasive wear of the enamel and dentine specimens after brushing with artificial saliva was minimal, that is, below the threshold for a potential measurement error of 0.1µm. Furthermore, the enamel specimens brushed with toothpaste exhibited negligible loss below the limit of detection. In contrast, dentine specimens brushed with toothpaste slurry showed a clearly detectable abrasive wear pattern, as shown in Figure 1. Wear was influenced by the IDBs as follows (in ascending order): Curaprox (median ± IQR: 8.6 ± 1.0 µm), Circum (9.7 ± 2.9 µm), and Elmex (18.8 ± 9.1 µm). The latter showed significantly more wear than the other two IDBs (P < 00.001); however, the difference in wear between Curaprox and Circum was not statistically significant (P = 1).

### SEM Observations

#### Enamel specimens

**Fig 2:**
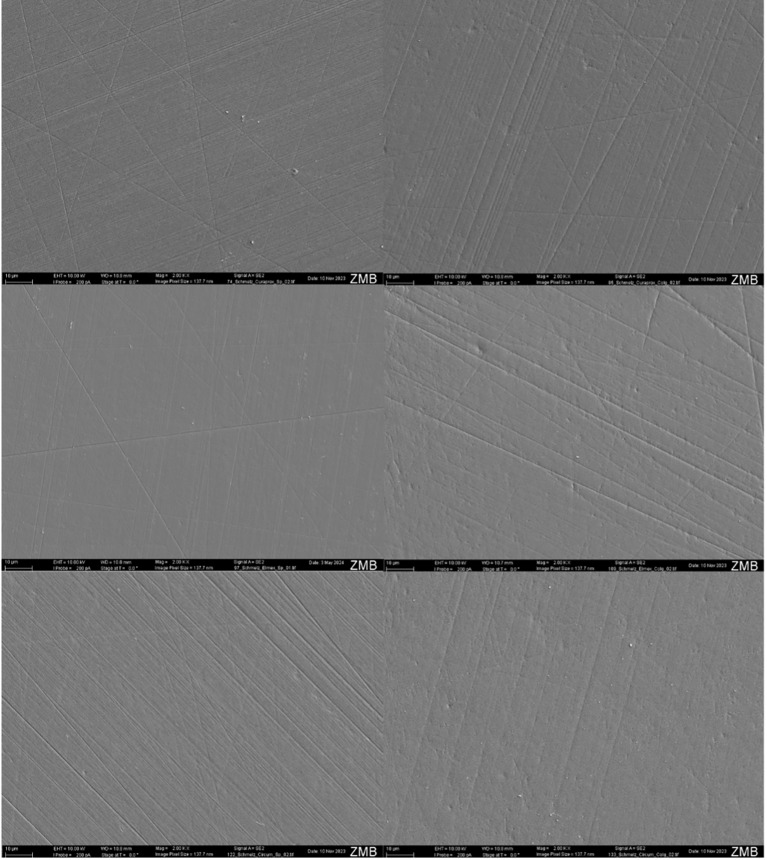
SEM images of enamel surfaces after 6 h of brushing, corresponding to 43,200 strokes in total. The specimens on the left were brushed using artificial saliva, whereas those on the right were brushed using toothpaste (Colgate Total Original). At the bottom is a reference specimen that has been polished but not brushed. The white line at the bottom left of each image corresponds to 10 µm.

The SEM images of the enamel specimens are presented in Figure 2.

Brushing with artificial saliva resulted in a smooth surface with thin scratches, some parallel and others transverse. A native specimen that underwent polishing but not brushing for comparison purposes exhibited only fine parallel indentations. Therefore, some notches were caused by the polishing process.

However, the notches on the specimens brushed with Curaprox and Elmex using Colgate Total Original appeared deeper and clearer, whereas the surfaces brushed with Curaprox and Circum using toothpaste showed greater topographical similarities.

#### Dentine specimens

**Fig 3:**
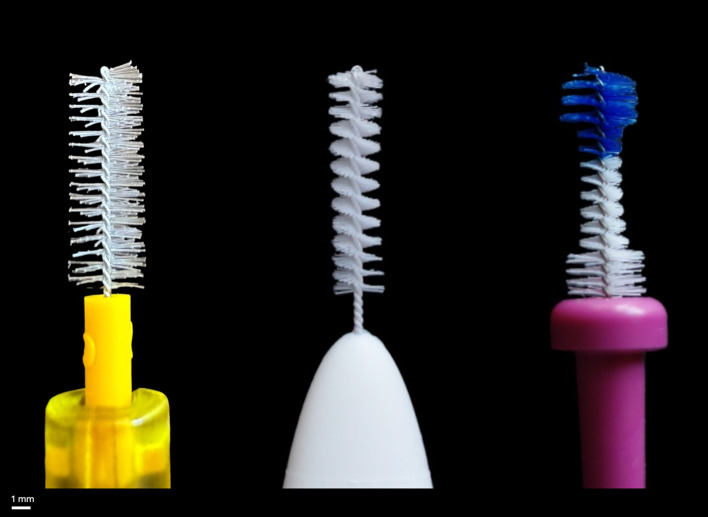
SEM images recorded dentine surfaces after 1 h of instrumentation (equivalent to 7,200 strokes) in the brushing machine. The specimens on the left were brushed using artificial saliva, whereas those on the right were brushed using toothpaste (Colgate Total). At the bottom is a reference specimen that has been polished but not brushed. The white line at the bottom left of each image corresponds to 10 µm.

SEM images of the dentine specimens are presented in Figure 3.

All of the dentine surfaces brushed with artificial saliva appeared homogeneous. Scratches resulting mainly from the polishing process were visible.

The surfaces brushed with Colgate Total Original showed clear parallel lines caused by the brushing process, which varied in extent. The dentine surface brushed with Elmex exhibited deeper and slightly wider notches. Some removal of the tooth structure in the Circum and Curaprox specimens was also visible in the form of parallel lines; however, the notches appeared slightly finer and less pronounced than those in the Elmex specimen.

The visibility of the dentine tubules varied between specimens. This may be a consequence of the cutting plane of the specimens and is not attributable to the different brushes used.

## DISCUSSION

During daily tooth brushing, approximately 60% of residual plaque is left, with the majority being left interdentally, followed by the oral and buccal surfaces.^7^ In recent years, dental prophylaxis has increasingly focused on interdental cleaning aids. A plethora of products are available that have varying degrees of efficiency. However, in this context, IDBs have been found to be more effective than most alternative oral hygiene aids.^20^ It is essential to note that improper use of interdental oral hygiene aids can also potentially lead to abrasive lesions. Unfortunately, there are no reliable data on the prevalence of exclusively abrasive lesions. A systematic review evaluated the prevalence of non-carious cervical lesions (NCCLs) and estimated that 46.7% of the population was affected, with a higher estimate in the older adult population.^24^

Currently, there is a paucity of studies and data on the adverse effects of proximal cleaning. Therefore, it is challenging to compare our results with those of previous studies. The results of the present study showed that dentine specimens brushed with toothpaste exhibited significant loss due to abrasion. The loss of dentine specimens brushed with saliva as well as enamel specimens, whether brushed with or without toothpaste, was negligible and insignificant. Thus, the null hypothesis that there would be no significantly higher loss of tooth structure when using IDBs with toothpaste than when using IDBs alone failed to be rejected for enamel specimens. These findings align with those of previous studies on the abrasive behaviour of toothbrushes and can be attributed, among other factors, to the varying hardness resulting from the different histological structures of dental hard tissues.^1,17^

Bovine specimens were used in this study. Research has indicated that bovine enamel and dentine specimens are not directly comparable to the human tooth structure. However, owing to their structural proximity, they offer a viable alternative in dental research.^3,26^ This study focused on enamel and dentine analyses. Exposure of the root cementum is a common phenomenon during periodontal recession. Therefore, further investigation of the abrasion behaviour of the root cementum could be beneficial.

Sound enamel and dentine were used, whereas *in vivo*, teeth are exposed daily to erosive influences that affect hardness and abrasion behaviour. Fluoride content and abrasive substances in toothpaste are relevant in this context. Eroded enamel wear caused by regular toothpaste was comparable to that of sound enamel, whereas eroded enamel showed less sensitivity to abrasion by diamond abrasives.^25,27^ Attin et al compared uneroded dentine with dentine treated with a high-concentration fluoride solution (2,000 ppm) following an erosive attack. These specimens exhibited similar abrasion behaviours.^4^ On the other hand, eroded dentine specimens treated with a low-concentration fluoride solution (250 ppm) exhibited significantly higher abrasion.^4^ According to the manufacturer, Colgate Total Original contains 1,450 ppm fluoride, which is in the upper middle range of the fluoride concentrations employed by Attin et al. Consequently, in real-life IDB use over extended periods, levels of dentine wear may exceed those observed in this laboratory study.

The manufacturers of the IDBs analysed recommend a simple ‘in-and-out’ technique but differ in the number of repetitions, ranging from one to four.^5,6,8^ The repetitive horizontal brushing of the laboratory set-up simulated approximately the recommended clinical handling. The brushing frequency for this study was 120 strokes per min, with 43,200 strokes for enamel and 7,200 strokes for dentine. As such, a worst-case scenario model was employed in this study by applying, from a clinical perspective, four strokes per day and site, which corresponded to an overall brushing period of 30 years for enamel and 5 years for dentine. The brushes were passed through a hole with a defined passage hole diameter (PHD) defined by ISO standards (ISO16409:2016). Because the IDBs were not manufactured exclusively for this study, it was to be expected that their diameters would deviate from the manufacturer’s specifications. This represents a limitation of the study but also corresponds with the clinical situation that the study intended to reflect.

The medium with which the specimens were brushed was related to the level of abrasion. The abrasiveness of toothpaste varies and can be assessed using the REA or RDA values. Studies have indicated that the REA values cannot be extrapolated from the RDA values of toothpaste.^28^ No REA or RDA information is available from the manufacturer of the Colgate Total Original toothpaste used in the study. Hamza et al found the mean and standard deviation of the RDA for Colgate Total Original to be 100 ± 5, classifying it as ‘very strongly abrasive’. In contrast, its REA value is considered ‘low abrasive’, with a value of 4 ± 2.11. *In-vitro* studies have shown a linear relationship between mean dentine abrasion and RDA values. However, this relationship is less evident *in vivo*.^17,18^ The fact that the Colgate Total Original toothpaste selected for the study had a very high RDA value but a low REA value could have influenced the results. Studies on tooth brushing on enamel have shown that an increased REA can significantly increase abrasion.^14^

Notably, our study used slurries, whereas in clinical settings toothpaste can be directly applied to the brush, bypassing the diluting effect of saliva. Consequently, the values obtained in our study potentially underestimated the abrasive damage. The specimens were stored in a slurry for the entire brushing cycle, which is a limitation of the study design. In clinical practice, remineralisation occurs after each brushing session. Furthermore, the IDBs did not change during the brushing period, which is not in accordance with the recommended clinical practice of changing IDBs after one to two weeks of daily use.^5^

Our findings indicate that toothpaste is crucial for determining the occurrence of hard substance loss. This is in accordance with prior studies showing that the extent of abrasive wear on sound tooth structure is significantly affected by the abrasiveness of the toothpaste used.^17^ The wear of dentine specimens brushed with different IDBs using toothpaste varied significantly. The null hypothesis, which anticipated comparable wear among the investigated IDBs, was rejected. This implies that the impact of IDB properties should also be considered.

The greatest wear of the dentine specimens brushed with toothpaste was caused by the Elmex IDB (substance loss: 18.8 ± 9.1 µm). The abrasion caused by the aforementioned IDB in comparison to Curaprox and Circum was statistically significant. Despite being categorised as ISO 2 according to the ISO standard for IDBs (ISO16409:2016), the three IDBs differ, as illustrated in Figure 4. Manufacturer specifications diverge, with most specifying the diameter of the IDB, followed by the diameter of the wire.^22^ A third of manufacturers state the ISO size.^22^ In the case of Curaprox (4 mm) and Circum (4 – 2 – 4 mm), the manufacturers state the diameter, with Elmex stating the diameter of the wire as 0.5 mm. The shapes of the two cylindrical IDBs from Curaprox and Elmex differ from the waist-shaped form of Circum. The filaments of Curaprox are longer, softer, and less densely arranged than those of Elmex and Circum. Notably, the bristles of Elmex are shorter but significantly harder than those of Curaprox and Circum. The significantly increased substance removal by Elmex IDBs suggests a potential correlation with the hardness of the bristles. The abrasive effects of toothbrushes on teeth varied depending on their bristle stiffness and the force applied.^12^ This finding could be further investigated for IDBs in a subsequent study.

## CONCLUSIONS

The improper use of IDBs, particularly long-term cleaning with toothpaste-dipped IDBs, may result in severe hard tissue loss. These findings demonstrate that dentine surfaces brushed with toothpaste exhibit abrasion. In addition, the properties of IDB influence the degree of abrasion. Therefore, providing accurate instructions to users is crucial to minimise the risk of abrasive damage.

### Acknowledgement

This study was conducted as a doctoral thesis by Med Dent Chiara Marconi and was performed at the Center for Dental Medicine in the Clinic of Conservative and Preventive Dentistry at the University of Zurich, Switzerland, under the supervision of Prof P.R. Schmidlin.

**Fig 4a to c:** The three ISO 2 normed IDBs used in the study: the cylindrical Curaprox (a), the cylindrical Elmex (b), and the waist-shaped Circum (c)


